# Long noncoding RNA LINC00518 acts as a competing endogenous RNA to promote the metastasis of malignant melanoma via miR-204-5p/AP1S2 axis

**DOI:** 10.1038/s41419-019-2090-3

**Published:** 2019-11-11

**Authors:** Wenkang Luan, Yuting Ding, Shaojun Ma, Hongru Ruan, Jinlong Wang, Feng Lu

**Affiliations:** 1 Department of Plastic Surgery, Affiliated People’s Hospital of Jiangsu University, Zhenjiang, Jiangsu China; 2 Department of Rehabilitation, Changshu No. 2 People’s Hospital (The 5th Clinical Medical College of Yangzhou University), Changshu, Jiangsu China

**Keywords:** Oncogenes, Melanoma, Cell invasion

## Abstract

Long intergenic nonprotein coding RNA 518 (LINC00518) has been shown to promote cancer cell growth and metastasis in some human tumors. Although it has been reported that LINC00518 is dysregulated in melanoma, its exact role and molecular mechanism in melanoma remain unclear. RNA-seq analysis and qRT-PCR was used to detect the expression of LINC00518 in melanoma tissues. Melanoma cases from The Cancer Genome Atlas (TCGA), GEO#GSE15605 and GEO#GSE24469 were included in this study. 3D migration, transwell and scratch wound assay were used to explore the role of LINC00518 in melanoma cells. Bioinformatics, luciferase reporter assays, MS2-RIP assay, RNA pull-down assay and RNA-ChIP assay were used to demonstrate the mechanism of LINC00518 in melanoma. We found that LICN00518 was significantly upregulated in melanoma tissue, and high LICN00518 level was an independent risk factor for melanoma patients. LICN00518 promoted the invasion and migration of melanoma cells. LICN00518 exerted its role by decoying miR-204-5p to upregulate Adaptor Related Protein Complex 1 Sigma 2 Subunit (AP1S2) expression. We also demonstrated that LICN00518 promoted melanoma metastasis in vivo through pulmonary metastasis assay. This result elucidates a new mechanism for LICN00518 in the metastasis of melanoma. LICN00518 may serve as a survival indicator and potential therapeutic target in melanoma patients.

## Introduction

Malignant melanoma is the leading cause of skin cancer-related death^1^. The global incidence of melanoma is increasing year by year^2^. Although melanoma therapy combines surgery, chemotherapy, targeted therapy and immunotherapy, its prognosis remains poor, especially in patients with distant metastasis^3^. The malignant progression of melanoma involves complex regulatory changes in multiple genes and signaling pathways^4,5^. Therefore, it is important to explore the molecular mechanisms behind melanoma progression and discover new specific biomarkers for melanoma.

Long noncoding RNA (lncRNAs) is a kind of noncoding RNA with a length of more than 200 nucleotides, which plays an important role in cancer biology^6,7^. Some lncRNAs can be used as the biomarker for the diagnosis and prognosis of numerous human tumors^8,9^. A few lncRNAs involved in the malignant progression of melanoma have been identified^10,11^. Long intergenic nonprotein coding RNA 518 (LINC00518), mapped to chromosome 6, has been shown to be upregulated and promote cancer cell growth and metastasis in breast and cervical cancer^12,13^. LINC00518 also contributes to chemotherapeutic drug resistance in breast and prostate cancer^14,15^. It was reported that LINC00518 is dysregulated in melanoma^16^. However, its exact role and molecular mechanism in melanoma remain undetermined.

Extensive studies have shown that certain specific lncRNAs may act as competitive endogenous RNAs (ceRNA) in tumorigenesis and development^17,18^. It has been demonstrated that LICN00518 plays the same role in prostate and breast cancer^14,15^. Here, we analyzed the expression profiles of lncRNAs in melanoma tissue, and constructed the ceRNA network of LICN00518 according to RNA-seq and miRNA-seq analysis results and bioinformatics predictions. We showed that LICN00518 was significantly upregulated in melanoma tissue, and high LICN00518 level was an independent risk factor for the prognosis of melanoma patients. LICN00518 promoted the invasion and migration of melanoma cells trough regulating Adaptor Related Protein Complex 1 Sigma 2 Subunit (AP1S2, a validated pro-motility target^19^). We also found that miR-204-5p exerts its antimotility activity in melanoma by targeting AP1S2, and LICN00518 could bind to miR-204-5p. Taken together, we concluded that LICN00518 act as a ceRNA to regulate AP1S2 expression by decoying miR-204-5p. Thus, LICN00518 can be used as a diagnostic and prognostic indicator and potential therapeutic in melanoma patients.

## Results

### LINC00518 was upregulated in melanoma tissues and cells

**Fig. 1:**
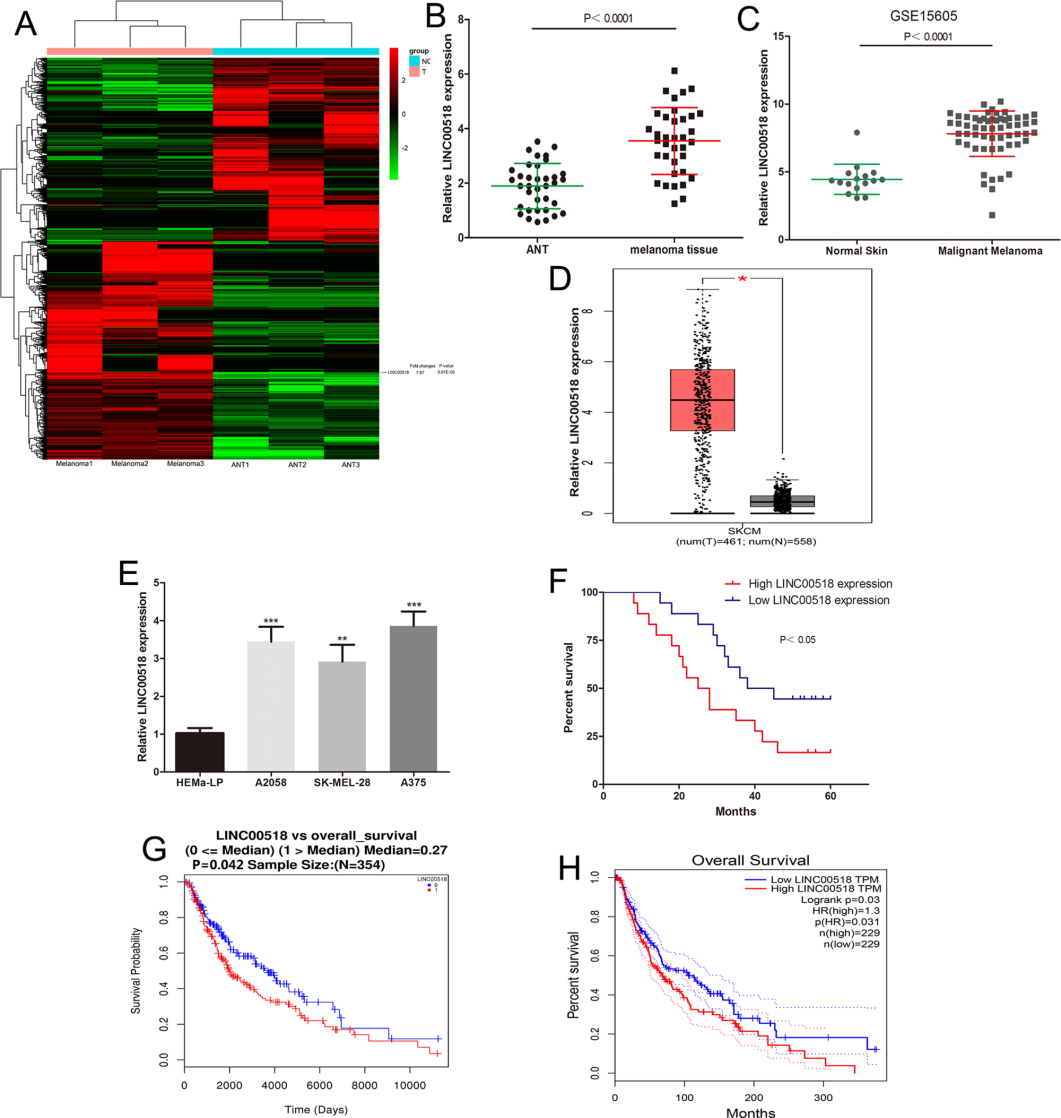
LINC00518 was upregulated in melanoma tissues and found to be a risk factor for the survival of melanoma patients. **a** The cluster heat map showed all differentially expressed lncRNAs over 2.5-fold change in melanoma. **b** The LINC00518 levels were detected in 36 malignant melanoma tissues and adjacent normal tissues. **c** The expression of LINC00518 was analyzed using GEO#GSE15605 dataset. **d** The expression of LINC00518 was detected from TCGA by using GEPIA (http://gepia.cancer-pku.cn/). **e** The LINC00518 expression profile in human melanoma cell lines (A375, A2058, SK-MEL-28) and human epidermal melanocytes (HEMa-LP). **f** The overall survival curves of 36 melanoma patients with high LINC00518 levels and low LINC00518 levels. **g** The prognostic data of melanoma in TCGA by using LinkedOmics (http://www.linkedomics.org). **h** We analyzed the prognostic data of melanoma in TCGA by using GEPIA (http://gepia.cancer-pku.cn/). Data were expressed as the mean ± SD, **P < 0.01, ***P < 0.001

RNA-seq analysis revealed lncRNA expression profiling in malignant melanoma tissues and ANT samples (three melanoma tissues and three ANT samples). The cluster heat map showed all differentially expressed lncRNAs over 2.5-fold change (Fig. 1a). The expression of LINC00518 in melanoma tissues was upregulated 7.67-fold (Fig. 1a). To verify the RNA-seq results, we analyzed LINC00518 levels in 36 melanoma tissues and ANT samples. LINC00518 was increased in melanoma tissues compared to ANT samples (Fig. 1b). We found the same result by analyzing the previously published dataset (GEO#GSE15605) (Fig. 1c). Then, we analyzed the expression profile of lncRNAs in melanoma from TCGA by using GEPIA (http://gepia.cancer-pku.cn/), and found that LINC00518 level was also upregulated in melanoma tissues (Fig. 1d). Malignant melanoma cell (A375, A2058, SK-MEL-28) expressed higher LINC00518 levels compared to human epidermal melanocytes (HEMa-LP) (Fig. 1e). Our results suggested that LINC00518 may be involved in the malignant progression of melanoma.

### Association between the expression of LINC00518 and prognosis in melanoma patients

We further explore the clinical significance of LINC00518 in melanoma. High LINC00518 levels (LINC00518 expression ratio ≥ median ratio) were associated with the clinical stage of melanoma, but not with age, sex, ulcer and family history (Table 1). In our patient samples, Kaplan−Meier analysis showed that melanoma patients with high LINC00518 levels had poorer survival (Fig. 1f). Meanwhile, we analyzed the prognostic data of melanoma in TCGA by using GEPIA (http://gepia.cancer-pku.cn/) and LinkedOmics (http://www.linkedomics.org), and found that overexpression of LINC00518 was correlated with poor survival of melanoma patients (Fig. 1g, h). More importantly, univariate and multivariate Cox regression analyses further demonstrated that high LINC00518 expression is an independent risk factor for the melanoma patients (Table 2).

**Table 1:** Correlation between LINC00518 levels and clinical pathological characteristic (*n* = 36)

Clinical characteristics	Number	High LINC00518 expression	Low LINC00518 expression	*P* value
Age				0.729
<50	13	6	7	
≥50	23	12	11	
Gender				0.735
Male	21	10	11	
Female	15	8	7	
Family history				0.074
Yes	6	5	1	
No	30	13	17	
TMN stage				<0.01
I-II	14	2	12	
III	22	16	6	
Ulcer				0.180
Yes	20	12	8	
No	16	6	10

**Table 2:** Univariate and multivariate Cox regression analysis of LINC00518 levels associated with overall survival rate in melanoma patients

Univariate analysis	Hazard ratio	95% CI	*P* value
LINC00518 expression (high vs. low)	2.538	1.115–5.774	0.026

Multivariate analysis	Hazard ratio	95% CI	*P* value
LINC00518 expression (high vs. low)	2.295	1.026–5.132	0.043

### ceRNA analysis for LINC00518

**Fig. 2:**
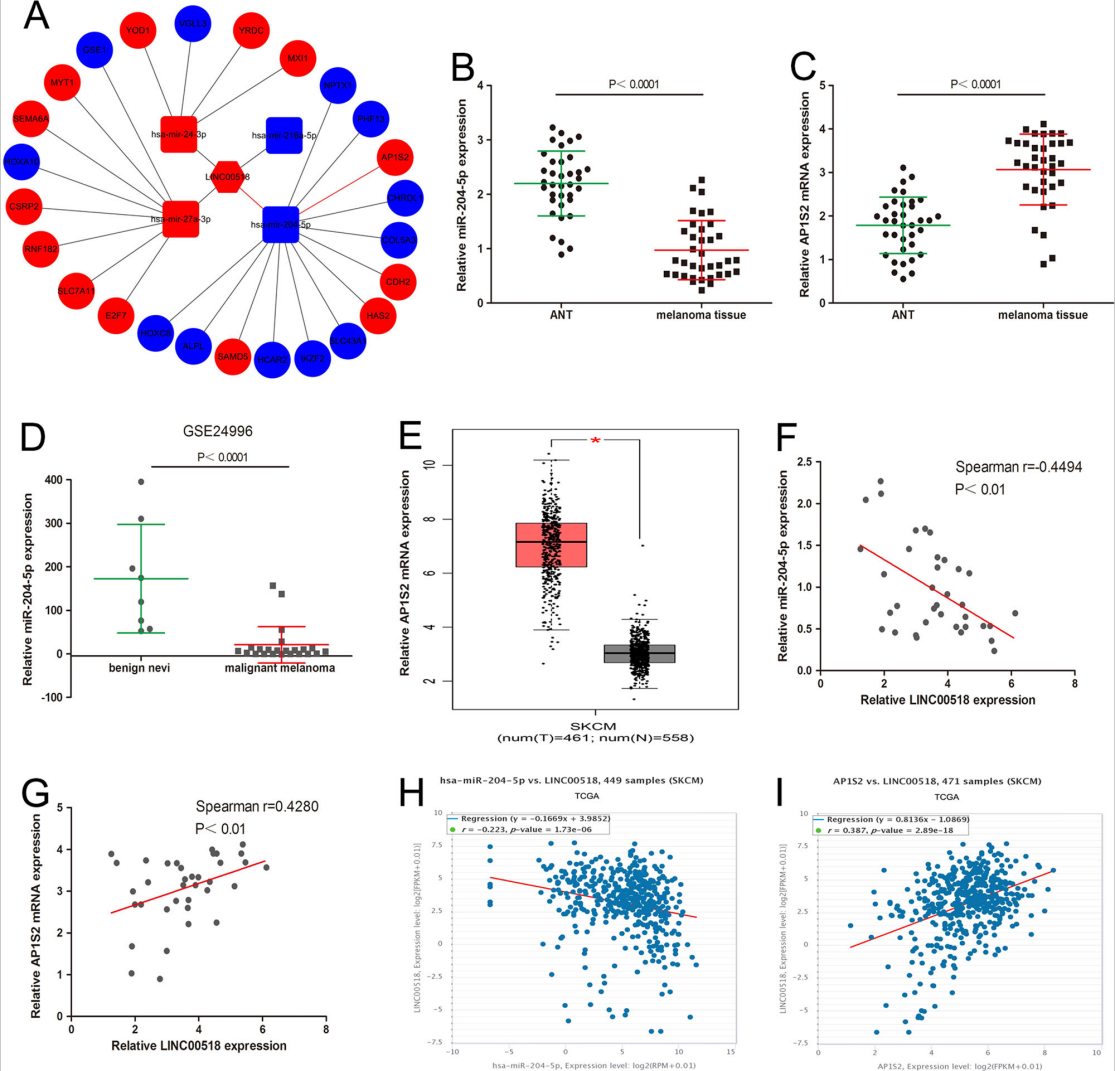
ceRNA analysis for LINC00518. **a** Cytoscape was used to visualize LINC00518−miRNA−target gene interactions based on our miRNA-seq and RNA-seq data. The LINC00518/miRNAs interaction was predicted using miRcode and DIANA LncBase Predicted, the target genes of the miRNAs were identified using TargetScan, miRDB and miRTarBase. Red color indicates high expression level, and blue color indicates low expression level. **b** The miR-204-5p levels were detected in 36 malignant melanoma tissues and adjacent normal tissues. **c** The expression of AP1S2 was analyzed in malignant melanoma tissues. **d** The expression of miR-204-5p was analyzed by using GEO#GSE24996 dataset. **e** The expression of AP1S2 was detected from TCGA by using GEPIA (http://gepia.cancer-pku.cn/). **f** The correlation of LINC00518 and miR-204-5p in 36 melanoma tissues was negative. **g** The positive correlation between LINC00518 and AP1S2 mRNA levels in 36 melanoma tissues. **h** TCGA melanoma dataset revealed a significant negative correlation between LINC00518 and miR-204-5p expression. **i** TCGA supports the positive correlation between LINC00518 and AP1S2 mRNA levels in melanoma. Data were expressed as the mean ± SD

LINC00518 plays a role in some human tumors through the ceRNA mechanism. Supposing that LINC00518 also has the same effect in melanoma, we constructed a LINC00518-miRNA-target gene network using Cytoscape to visualize their interrelationships based on our miRNA-seq and RNA-seq data (Fig. 2a). The LINC00518/miRNAs interaction was predicted using miRcode and DIANA LncBase Predicted, the target genes of the miRNAs were identified using TargetScan, miRDB and miRTarBase. In the network, miR-204-5p captured our attention because of its expression was downregulated in melanoma tissue (Fig. 2a). AP1S2 (a validated pro-motility target^19^), the predicted target of miR-204-5p, was increased in melanoma tissue (Fig. 2a).

### Interrelationship among LINC00518, miR-204-5p and AP1S2 in melanoma

To validate the results of ceRNA analysis, the expression of miR-204-5p and AP1S2 was measured in 36 melanoma tissues and ANT samples. miR-204-5p was decreased and AP1S2 was increased in melanoma tissues (Fig. 2b, c). The same result is obtained by analyzing the previously published dataset (GEO#GSE24996) and TCGA database (Fig. 2d, e). Meanwhile, we found a negative correlation between LINC00518 and miR-204-5p levels, and a positive correlation between LINC00518 and AP1S2 mRNA levels in 36 melanoma tissues (Fig. 2f, g). The analysis of TCGA datasets also supports the above correlation analysis results (Fig. 2h, i).

### LINC00518 serves as a sponge for the miR-204-5p in melanoma

**Fig. 3:**
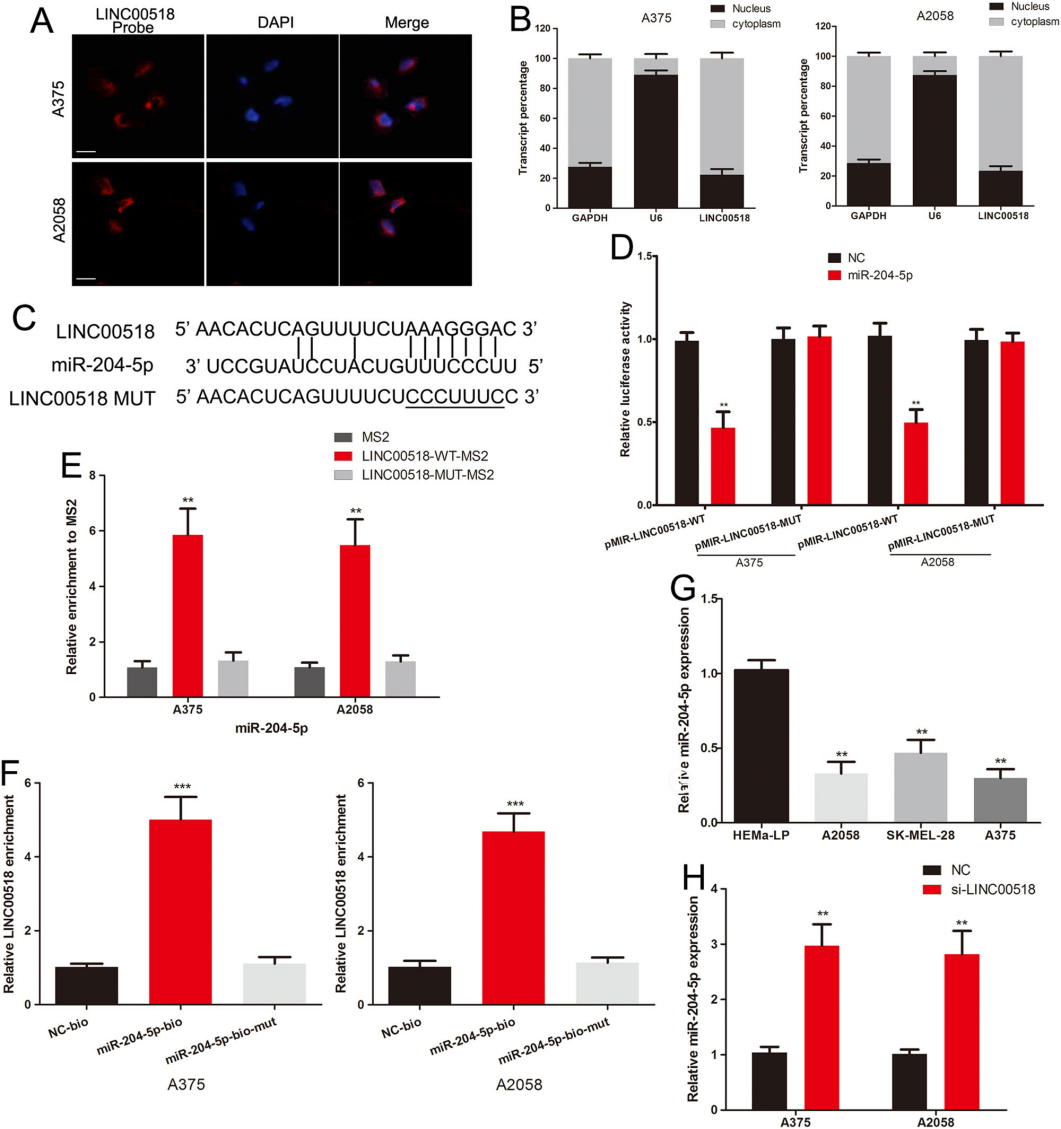
LINC00518 serves as a sponge for the miR-204-5p in melanoma. **a** FISH revealed that LINC00518 was mainly distributed in cytoplasm in melanoma cells. Scale bar, 25 μm. **b** qRT-PCR assays in nuclear and cytoplasmic RNA fractions detected the LINC00518 level in cytoplasm and nuclear. **c** The putative binding sites of miR-204-5p on LINC00518, and target sequences were mutated. **d** Luciferase assay of melanoma cells transfected with LINC00518-WT or LINC00518-MUT reporter together with miR-204-5p or NC. **e** MS2-RIP followed by miRNA PCR to detect endogenous miR-204-5p associated with the MS2-tagged LINC00518. **f** Melanoma cells transfected with biotin-labeled miR-204-5p, mutated oligos or NC, assayed by biotin-based pull down. LINC00518 levels were analyzed by qRT-PCR. **g** The miR-204-5p expression profile in human melanoma cell lines (A375, A2058, SK-MEL-28) and human epidermal melanocytes (HEMa-LP). **h** The expression levels of miR-204-5p in melanoma cells following transfection with LINC00518 siRNA or NC. Data were expressed as the mean ± SD, **P < 0.01, ***P < 0.001

We further explore the potential interaction between LINC00518 and miR-204-5p. FISH and qRT-PCR of nucleus and cytoplasm fractions revealed that LINC00518 was mainly localized in cytoplasm in melanoma cells (Fig. 3a, b). We next constructed LINC00518 luciferase plasmids containing the wild-type and mutant miR-204-5p binding sites (Fig. 3c). miR-204-5p mimics significantly inhibited the luciferase activity of wild-type LINC00518 vector in melanoma cells, but not that of the mutant plasmid (Fig. 3d). MS2-RIP and RNA pull-down assay was used to further verify the direct interaction between miR-204-5p and LINC00518. The MS2-tagged wild-type LINC00518 vector was enriched for miR-204-5p compared to the empty and mutant plasmids (Fig. 3e). Moreover, RNA pull-down assay with biotinylated miR-204-5p found that LINC00518 was pulled down by biotin-labeled miR-204-5p (Fig. 3f). The expression of miR-204-5p was decreased in malignant melanoma cell (Fig. 3g). Meanwhile, miR-204-5p was overexpressed after transfection of LINC00518 siRNA in A375 and A2058 cells (Fig. 3h). All results showed that LINC00518 directly binds to miR-204-5p in melanoma.

### LINC00518 promotes AP1S2 expression through sponging miR-204-5p

**Fig. 4:**
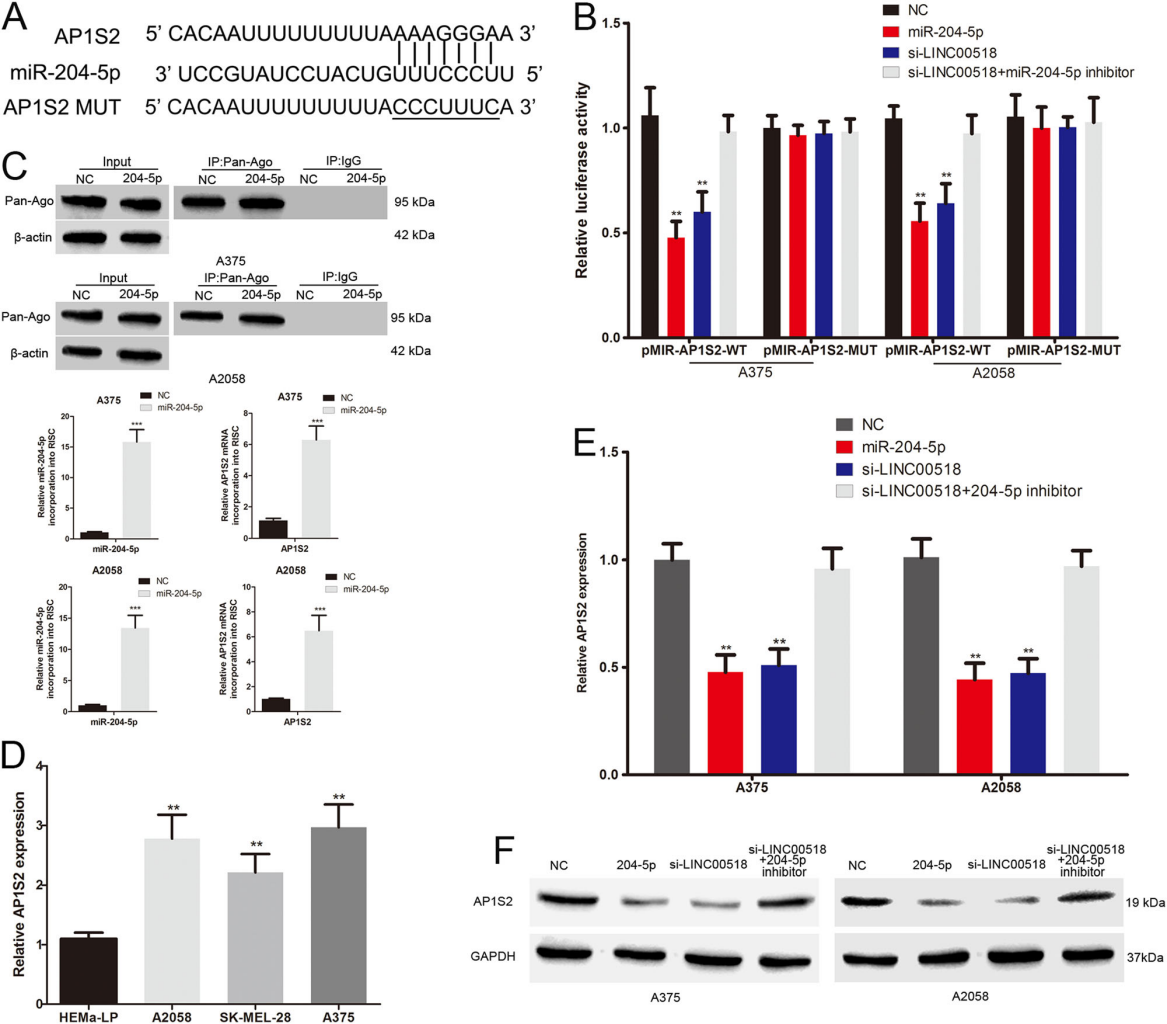
LINC00518 promotes AP1S2 expression through sponging miR-204-5p. **a** The binding sites of miR-204-5p on the 3′-UTR of AP1S2. Target sequences of AP1S2-3′ UTRs were mutated. **b** Luciferase assay of cells transfected with AP1S2-3′ UTR-WT or AP1S2-3′ UTR-MUT reporter together with miR-224-5p, LINC00518 siRNA or LINC00518 siRNA plus miR-204-5p inhibitor. **c** Immunoprecipitation of the Ago2/RISC using the Pan-Ago2 antibody in melanoma cells overexpressing miR-204-5p. IgG was used as a negative control and β-actin was used as an internal control. PCR analysis of miR-204-5p and AP1S2 incorporated into RISC in melanoma cells overexpressing miR-204-5p. **d** The AP1S2 expression profile in human melanoma cell lines (A375, A2058, SK-MEL-28) and human epidermal melanocytes (HEMa-LP). **e** The expression of AP1S2 mRNA in melanoma cells transfected with miR-224-5p, LINC00518 siRNA or LINC00518 siRNA plus miR-204-5p inhibitor. **f** Western blots identified AP1S2 protein expression changes following transfection with miR-224-5p, LINC00518 siRNA or LINC00518 siRNA plus miR-204-5p inhibitor, GAPDH was used as a control. Data were expressed as the mean ± SD, **P < 0.01, ***P < 0.001

The 3′-UTR of AP1S2 has the same binding sites that LINC00518 combined with miR-204-5p (Fig. 4a). Overexpression of miR-204-5p led to a marked decrease in luciferase activity of the wild-type AP1S2 3′UTR vector (Fig. 4b). We next detected the AP1S2 mRNA abundance in the Ago2/RNA-induced silencing complex (RISC) after overexpression of miR-204-5p by using RNA-ChIP analysis (Fig. 4c). Enrichment in the level of miR-204-5p and AP1S2 that incorporated into RISC was observed in miR-224-5p mimic transfected cells (Fig. 4c). The expression of AP1S2 was increased in malignant melanoma cell (Fig. 4d). miR-204-5p mimics also repressed the level of AP1S2 mRNA and protein in melanoma cells (Fig. 4e, f). These results indicated that AP1S2 is the target gene of miR-204-5p. Moreover, the luciferase activity of the wild-type AP1S2 3′UTR plasmid was significantly decreased by LINC00518 siRNA, and this effect can be reversed by miR-204-5p inhibitor (Fig. 4b). LINC00518 siRNA also led to a decrease in the mRNA and protein levels of AP1S2 in melanoma cells, and this inhibition was reversed by cotransfection with miR-204-5p inhibitor (Fig. 4e, f). In conclusion, these results suggested that LINC00518 promotes AP1S2 expression by competitive binding miR-204-5p in melanoma.

### LINC00518 promotes the melanoma cell invasion and migration through miR-204-5p/AP1S2 axis

**Fig. 5:**
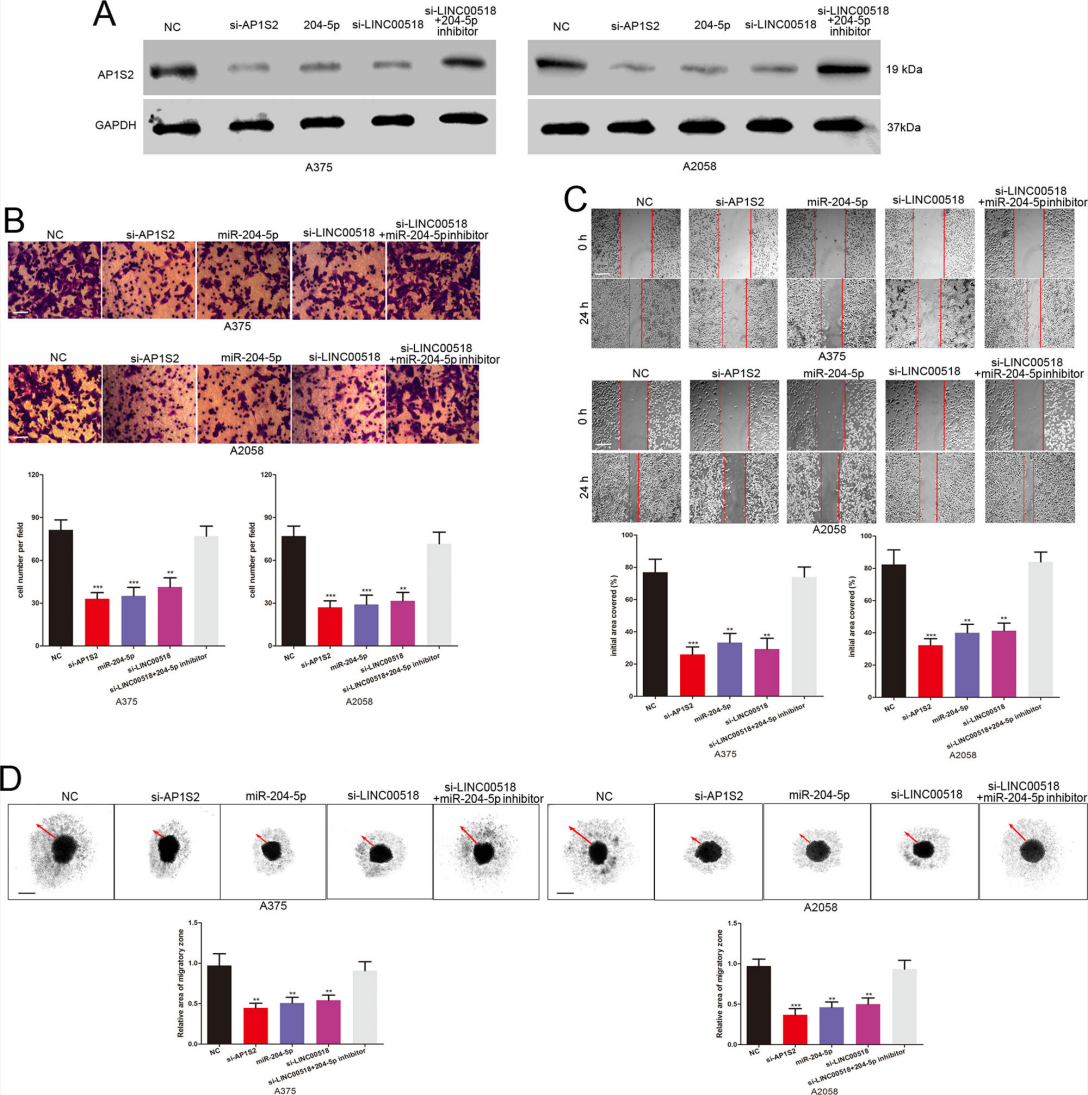
LINC00518 promotes the melanoma cell invasion and migration through miR-204-5p/AP1S2 axis. **a** Western blots identified AP1S2 protein expression changes in LINC00518 siRNA, AP1S2 siRNA, miR-204-5p mimic, LINC00518 siRNA plus miR-204-5p inhibitor transfected melanoma cells. GAPDH was used as a control. **b** The invasive capacity of LINC00518 siRNA, AP1S2 siRNA, miR-204-5p mimic, LINC00518 siRNA plus miR-204-5p inhibitor transfected melanoma cells was assessed by the transwell assay. Scale bar, 50 μm. **c** Migration of melanoma cells in different transfection groups was monitored by the scratch wound assay. Scale bar, 200 μm. **d** Results of melanoma cell migration were validated by a 3D migration assays. Scale bar, 200 μm. Data were expressed as the mean ± SD, **P < 0.01, ***P < 0.001

AP1S2, a validated pro-motility target, has been shown to facilitate the migration and invasion of melanoma cells^19,20^. We further explore the biological role of LINC00518 in melanoma cells. Stably expressing LINC00518 shRNA A375 cells showed decreased invasive and migratory ability, but no statistically significant change in growth and apoptosis (Supplementary Fig. 1A-D). LINC00518 siRNA, AP1S2 siRNA, miR-204-5p mimic, LINC00518 siRNA together with miR-204-5p inhibitor were transfected into A375 and A2058 cells, and western blotting was used to detect AP1S2 expression (Fig. 5a). Scratch wound assays and transwell assays showed that knockdown of AP1S2 and LINC00518 and overexpression of miR-204-5p significantly repressed the invasive and migratory ability of melanoma cells (Fig. 5b, c). Simultaneously, LINC00518 siRNA, AP1S2 siRNA and miR-204-5p mimic inhibited melanoma cell infiltration in a 3D collagen matrix, these melanoma cells showed a less invasive morphology (Fig. 5d). Moreover, the inhibitory effect of LINC00518 siRNA on the invasion, migration and infiltration of melanoma cells was attenuated by miR-204-5p inhibitor (Fig. 5b−d). Overall, it demonstrated that LINC00518 modulates metastasis of melanoma cells by sponging miR-204-5p to promote AP1S2 expression.

### LINC00518 exerts its prometastasis activity through regulating the AP1S2 levels in vivo

**Fig. 6:**
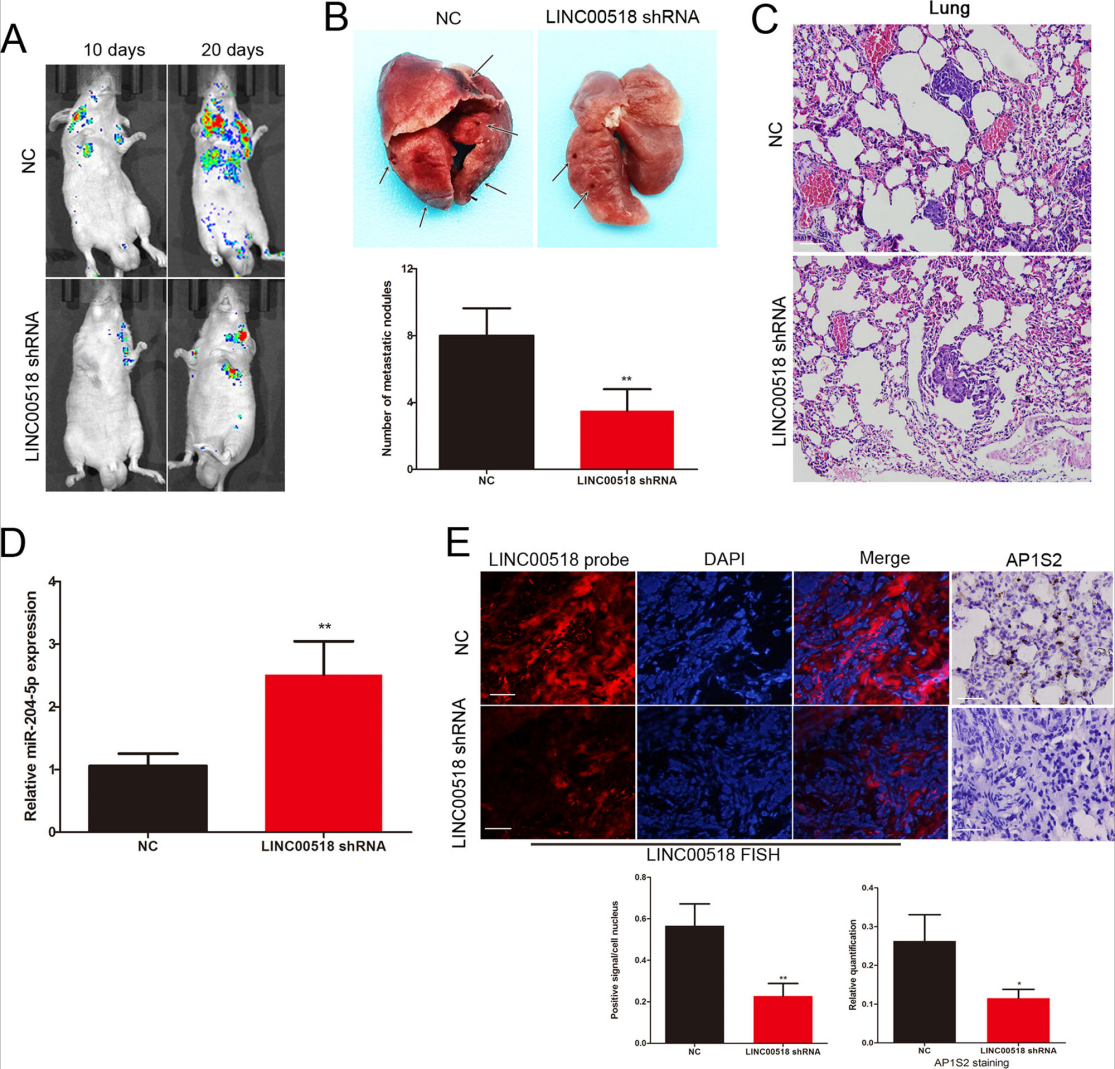
LINC00518 exerts its prometastasis activity through regulating the AP1S2 levels in vivo. **a** Representative bioluminescence images of mice after tail vein injection of stably expressing lentiviral control or LINC00518 shRNA A375 cells. **b** The excision lung tissues in nude mice and the number of metastatic lung nodules. **c** Metastatic lung nodules were confirmed by HE staining. Scale bar, 50 μm. **d** PCR identified miR-204-5p expression changes. **e** The expression of LINC00518 and AP1S2 were detected by FISH and immunohistochemistry of sections from the metastatic lung nodules. Scale bar, 25 μm. Data were expressed as the mean ± SD,*P < 0.05, **P < 0.01,

We investigated the effect of LINC00518 on the metastasis of melanoma cells in vivo. Stably expressing LINC00518 shRNA or miR-204-5p A375 cells were tail vein injected into nude mice. Silencing of LINC00518 in A375 cell displayed fewer lung colonization compared to the control group (Fig. 6a). Knockdown of LINC00518 also decreased the number of metastatic lung nodules (Fig. 6b). HE staining confirmed the tumor tissues from metastatic lung nodules (Fig. 6c). miR-204-5p-overexpressing A375 cells also displayed lower levels of lung colonization and fewer metastatic lung nodules (Supplementary Fig. 2A, B). We detected the expression of LINC00518, miR-204-5p and AP1S2 on the sections of metastatic lung nodules. The miR-204-5p level was increased with the knockdown of LINC00518 (Fig. 6d). The AP1S2 was decreased in the miR-204-5p overexpression group and LINC00518 knockdown group (Supplementary Fig. 2C, D, Fig. 6e). These results indicated that LINC00518 promotes melanoma metastasis in vivo through AP1S2.

## Discussion

Numerous studies have confirmed that lncRNAs play a key role in the malignant progression of many human tumors^18,21,22^. LINC00518, a newly identified lncRNA, has been reportedly to be upregulated in some malignant tumors^12,14^. LINC00518 contributes to the multidrug resistance of breast cancer through the miR-199a/MRP1 axis^14^. Wang et al. found that LINC00518 promotes the proliferation and metastasis of cervical cancer by modulating JAK/STAT3 pathway^12^. LINC00518 also promotes the paclitaxel resistance through targeting miR-216b-5p to regulate GATA6 expression in prostate cancer^15^. Although some studies have reported that LINC00518 is dysregulated in melanoma^16,23^, the exact function and mechanism of LINC00518 in melanoma are still unclear. Here, we analyzed the expression profile of lncRNAs in melanoma tissue. LINC00518 was found to be increased in melanoma and conferred a poor prognosis to melanoma patients based on our samples analysis and public database. LINC00518 also increased the invasion, migration and infiltration ability of melanoma cell.

Extensive studies have shown that lncRNAs may act as ceRNAs in carcinogenesis. ceRNAs can sponge miRNAs and reduce the binding of miRNAs to their target genes, thereby regulating gene expression^24^. LINC00518 also plays the same role in breast and prostate cancer^14,15^. We subsequently constructed the ceRNA network of LICN00518 according to RNA-seq and miRNA-seq analysis and bioinformatics predictions. Thirty-six melanoma tissues and public databases were used to validate the results of ceRNA analysis. LINC00518, miR-204-5p and AP1S2 were found to have underlying correlation in melanoma. Luciferase reporter assay, MS2-RIP assay and RNA pull-down assay showed that LINC00518 directly binds to miR-204-5p. We also demonstrated that AP1S2 is a direct target of miR-204-5p in melanoma. LINC00518 siRNA inhibited the mRNA and protein levels of AP1S2, and the luciferase activity of the wild-type AP1S2 plasmid. This inhibitory effect was reversed by miR-204-5p inhibitor. These results indicated that LINC00518 promotes AP1S2 expression by competitive binding miR-204-5p in melanoma.

Accumulating evidence has shown that miR-204-5p is downregulated and plays a role of tumor suppressor in many tumors, including melanoma^25–27^. For instance, miR-204-5p can inhibit the growth and invasion and enhances chemosensitivity of colorectal cancer by targeting RAB22A^28^. Vitiello et al. found that miR-204-5p acts as an effecter of vemurafenib’s antimotility activity by regulating AP1S2 in melanoma^19^. AP1S2 is a validated target for promoting metastasis of melanoma cells^19,20^. In this study, we further confirmed the antimotility role of miR-204-5p and the pro-motility role of AP1S2 in melanoma. Moreover, the effect of LINC00518 siRNA on melanoma cells can be rescued by cotransfection miR-204-5p inhibitor. These revealed that LINC00518 modulates the metastasis of melanoma cells through sponging miR-204-5p to promote AP1S2 expression. Lastly, we also showed that LINC00518 also promotes melanoma metastasis in vivo by regulating the expression of AP1S2.

In conclusion, we demonstrated that LINC00518 plays a key role in the malignant progression of melanoma. LINC00518 facilitates melanoma metastasis by sponging miR-204-5p to release AP1S2 mRNA transcripts targeted by miR-204-5p, thereby promoting the AP1S2 expression. Studying the molecular mechanism of LINC00518 in melanoma is of great significance to improve the understanding of the molecular biological basis of melanoma development and identify new therapeutic targets for melanoma patients.

## Materials and methods

### Patients and tissue samples

Thirty-nine primary malignant melanoma tissues and adjacent normal tissues (ANT) were obtained from the Affiliated People’s Hospital of Jiangsu University, and no patients received chemotherapy or radiotherapy before surgery. The histological characteristics of tissue were independently diagnosed by two pathologists. The study was approved by the Human Research Ethics Committee of the Affiliated People’s Hospital of Jiangsu University. Informed consent was obtained from all patients. Melanoma sample from The Cancer Genome Atlas (TCGA), GEO#GSE15605 and GEO#GSE24469 were also included in this study.

### RNA-seq, miRNA-seq and ceRNA analysis

Three melanoma tissues and three ANT samples were stored in liquid nitrogen immediately after collection. TRIzol reagent (Invitrogen, USA) was used for total RNA isolation. RNA-seq and miRNA-seq analysis were conducted by Gminix (Shanghai, China). We constructed a LICN00518-miRNA-target gene network using Cytoscape (v.3.6.0) to visualize their interactions based on our RNA-seq and miRNA-seq data. The LINC00518/miRNAs interaction was predicted using miRcode and DIANA LncBase Predicted, the target genes of the miRNAs were identified using TargetScan, miRDB and miRTarBase.

### Cell culture

The human malignant melanoma cell lines A375, A2058 and SK-MEL-28 were purchased from the American Type Culture Collection (ATCC), and human epidermal melanocytes HEMa-LP was obtained from Invitrogen. Melanoma cells were cultured in Dulbecco’s modified Eagle’s medium (DMEM; Gibco, USA) with 10% fetal bovine serum (Invitrogen, USA), epidermal melanocytes were grown in medium 254 (Cascade Biologics, USA). All cells were maintained in an atmosphere of 37 °C with 5% CO_2_.

### Oligonucleotides and transfection

miR-204-5p mimic, miR-204-5p inhibitor and related negative control (NC) were bought from GenePharma (Shanghai, China). LINC00518 small interfering RNA (siRNA) and short hairpin RNA (shRNA) and AP1S2 siRNA were chemically synthesized by GenePharma (Shanghai, China). The shRNA and its corresponding control sequences were inserted into the lentivirus vector (GenePharma, Shanghai, China). Melanoma cells were infected with lentiviruses in order to obtain stably expressing miR-204-5p or LINC00518 shRNA cells. The oligonucleotides were transfected into melanoma cells using Lipofectamine 3000 (Invitrogen, USA).

### Quantitative RT-PCR

RNA was extracted from cells and tissues using TRIzol reagent (Invitrogen, USA). Different reverse transcription kits (Applied Biosystems, CA) were used for reverse transcription. The StepOnePlus system (Applied Biosystems, CA) was used for amplification reactions according to the set reaction conditions. To detect the miR-204-5p level, the special primer was obtained from RiboBio (Guangzhou, China), and U6 was used for normalization. For analysis the LINC00518 and AP1S2 level, GAPDH was used for normalization. The following primers were used: LINC00518 forward 5′-GTGAAAATCTGGCTACTCGTCCC-3′ and LINC00518 reverse 5′-CTGACTTTTGCCACAGACTCCTG-3′; AP1S2 forward 5′-CCTTGAGTGGCGAGATCTGA-3′ and AP1S2 reverse 5′-CCTGATCCTCAATAGCACAGC′; GAPDH forward 5′- GTCAACGGATTTGGTCTGTATT-3′ and GAPDH reverse 5′- AGTCTTCTGGGTGGCAGTGAT-3′. The relative expression level was calculated using the 2^–△△Ct^.

### Western blot

Total proteins were extracted by using RIPA buffer (KenGEN, China). The extracted protein concentration was quantified with a BCA Protein Assay Kit (Beyotime, China). Western blotting is performed as previously described^29^. Antibodies against AP1S2 (Abcam, 1:1000, Cambridgeshire, UK) was used to analyze AP1S2 levels, β-actin (1:1000, Abcam, UK) and GAPDH (1:2500, Abcam, UK) were also used.

### Cell invasion and migration assays

For transwell assay, melanoma cells were resuspended in serum-free culture medium after transfected. Melanoma cells were then placed at the top of the Matrigel-coated chambers (BD Biosciences, USA). The medium containing 10% fetal bovine serum was added to the lower chamber as chemical attractant. After 24 h of culture, the invasive cells were stained with crystal violet and counted. For scratch wound assay, melanoma cells were added into six-well plates after transfected. The wound gap was formed by using 200 μl pipette tip. Cells were photographed and wound width was recorded at 0 and 24 h.

### 3D migration assays

Cell suspension droplet containing 1000 cells was placed on culture dish. Lids were inverted over dishes. Cell aggregates were collected and cultured of hanging drops for 48 h, and then implanted into three-dimensional collagen I gels (PureCol, Inamed, Fremont, CA, USA). We adjusted the pH of collagen I gels to 7.5 and supplemented collagen I gels with DMEM containing 2% fetal bovine serum. After polymerization at 37 °C, the collagen I gel was overlaid with DMEM containing 10% fetal bovine serum. Olympus microscope system was used to evaluate the result.

### Luciferase reporter assay

The fragment of AP1S2 3′-UTR and LINC00518 containing the binding site of miR-204-5p were inserted into the pMIR-REPORT vector. Melanoma cells were cotransfected with related oligonucleotides and luciferase reporter plasmids. Plasmids with mutation-binding sites were used as controls. Dual Luciferase Reporter Assay System (Promega, USA) was used to detect the luciferase activity of reporter plasmids.

### Isolation of RISC-associated RNA

We fixed melanoma cells that overexpressed miR-204-5p or miR-NC using 1% formaldehyde. Chromatin fragmentation is then processed. The cells were lysed using NETN buffer, and cultured with Dynabeads Protein A (Invitrogen, USA) supplemented with IgG or anti-Pan-Ago, clone 2A8 antibody (Millipore, USA). Proteinase K digestion was used to release immunoprecipitated RNA, and phenol/chloroform/isopropyl alcohol was used to extract RNA. RNA was purified by ethanol precipitation with glycogen and treated with DNase I.

### Fluorescence in situ hybridization (FISH)

FISH was carried out by using RiboTM Fluorescent In Situ Hybridization Kit (RiboBio, Guangzhou, China) and procedures were performed based on a previous study^30^. The LINC00518 probe was designed and synthesized by RiboBio (Guangzhou, China). Nucleus was stained with DAPI. The image was obtained by confocal microscope. ImageJ software was used to collect signals. Three visual fields were selected for each group.

### MS2-RIP assay

We used Maltose-binding protein (MBP)-affinity purification to identify miRNAs that associated with LINC00518. The MS2-MBP was expressed and purified from *Escherichia coli* according to Steitz laboratory steps. Three bacteriophage MS2 coat protein-binding sites were inserted downstream of LINC00518 by site-directed mutagenesis using Stratagene Quik Change Site Directed Mutagenesis Kit. Melanoma cell lines were transfected with MS2-tagged LINC00518 to obtain miRNAs associated with the LINC00518. At 48 h after transfection, the cells were subjected to RIP analysis as previously described^17^. The level of miR-204-5p was detected by qRT-PCR.

### RNA pull-down assay

Biotinylated miR-204-5p was bought from GenePharma (Shanghai, China). Biotinylated mutant and biotinylated NC were also synthesized and used as control. We transfected biotinylated oligonucleotides into melanoma cells. The cell lysates were cultured with M-280 streptavidin magnetic beads (Invitrogen, USA)^31^. The bound RNA was extracted and the level of LINC00518 was detected by qRT-PCR.

### In vivo tumor pulmonary metastasis assay

Twelve nude mice were purchased from the Beijing Laboratory Animal Center (Beijing, China). A375 cells stably expressing miR-204-5p or LINC00518 shRNA were injected into the tail vein of mice. Mice were intraperitoneal injected with 10 μl/g sterile d-Luciferin firefly potassium salt (Beyotime, China). PerkinElmer IVIS Spectrum (Xenogen, CA) was used for in vivo imaging. The results were quantified as the average radiance of photons emitted using the Living Image software (Xenogen, CA). The lungs were dissected and the metastatic nodules were counted after 20 days. The study was approved by the Experimental Animal Ethics Committee of the Affiliated People’s Hospital of Jiangsu University.

### Immunohistochemistry staining and HE staining

The process of immunohistochemistry is carried out as described previously^32^ using antibody against AP1S2. ImageJ software was used to analyze the optical density of the image. Three visual fields were selected for each group. For HE staining, sections were incubated with hematoxylin after deparaffinization and rehydration and stained with five dips in acid ethanol and eosin. The sections were dehydrated with graded alcohol and cleared in xylene. The images were taken by fluorescence microscope.

### Statistical analysis

The data are expressed as the mean ± standard deviation and analyzed by SPSS13.0. The statistical significance of the data was evaluated by *t* test or one-way ANOVA. Spearman correlation analysis was performed by using MATLAB. Survival plots were drawn based on Kaplan−Meier analysis. *P* < 0.05 was considered to have statistical significance.

## Supplementary information

Experimental Method of Supplementary Materials

Supplementary Figure 1

Supplementary Figure 2

## References

[1] Haass NK, Schumacher U (2014). Melanoma never says die. Exp. Dermatol..

[2] Little EG, Eide MJ (2012). Update on the current state of melanoma incidence. Dermatologic Clin..

[3] Tsao H, Chin L, Garraway LA, Fisher DE (2012). Melanoma: from mutations to medicine. Genes Dev..

[4] Paluncic J (2016). Roads to melanoma: key pathways and emerging players in melanoma progression and oncogenic signaling. Biochim. et Biophys. Acta.

[5] Uzdensky AB, Demyanenko SV, Bibov MY (2013). Signal transduction in human cutaneous melanoma and target drugs. Curr. Cancer Drug Targets.

[6] Hauptman N, Glavac D (2013). Long non-coding RNA in cancer. Int. J. Mol. Sci..

[7] Wang KC, Chang HY (2011). Molecular mechanisms of long noncoding RNAs. Mol. Cell.

[8] Gupta RA (2010). Long non-coding RNA HOTAIR reprograms chromatin state to promote cancer metastasis. Nature.

[9] Lv M (2017). lncRNA H19 regulates epithelial-mesenchymal transition and metastasis of bladder cancer by miR-29b-3p as competing endogenous RNA. Biochim. et Biophys. Acta Mol. Cell Res..

[10] Li R (2016). Long non-coding RNA BANCR promotes proliferation in malignant melanoma by regulating MAPK pathway activation. PLoS ONE.

[11] Luan W (2018). Long non-coding RNA H19 promotes glucose metabolism and cell growth in malignant melanoma via miR-106a-5p/E2F3 axis. J. Cancer Res. Clin. Oncol..

[12] Wang DW, You D, Dong J, Liu TF (2019). Knockdown of long non-coding RNA LINC00518 inhibits cervical cancer proliferation and metastasis by modulating JAK/STAT3 signaling. Eur. Rev. Med. Pharmacol. Sci..

[13] Yang F (2016). Co-expression networks revealed potential core lncRNAs in the triple-negative breast cancer. Gene.

[14] Chang L, Hu Z, Zhou Z, Zhang H (2018). Linc00518 contributes to multidrug resistance through regulating the MiR-199a/MRP1 axis in breast cancer. Cell. Physiol. Biochem.: Int. J. Exp. Cell. Physiol., Biochem., Pharmacol..

[15] He J, Sun M, Geng H, Tian S (2019). Long non-coding RNA Linc00518 promotes paclitaxel resistance of the human prostate cancer by sequestering miR-216b-5p. Biol. Cell.

[16] Ferris LK (2017). Utility of a noninvasive 2-gene molecular assay for cutaneous melanoma and effect on the decision to biopsy. Jama Dermatol..

[17] Luan W (2016). Long non-coding RNA MALAT1 acts as a competing endogenous RNA to promote malignant melanoma growth and metastasis by sponging miR-22. Oncotarget.

[18] Liu L (2018). Long non-coding RNA HOTAIR acts as a competing endogenous RNA to promote glioma progression by sponging miR-126-5p. J. Cell. Physiol..

[19] Vitiello M (2017). Context-dependent miR-204 and miR-211 affect the biological properties of amelanotic and melanotic melanoma cells. Oncotarget.

[20] Vitiello M, D’Aurizio R, Poliseno L (2018). Biological role of miR-204 and miR-211 in melanoma. Oncoscience.

[21] Zhang H (2013). Long non-coding RNA: a new player in cancer. J. Hematol. Oncol..

[22] Liu L (2019). The long non-coding RNA SNHG1 promotes glioma progression by competitively binding to miR-194 to regulate PHLDA1 expression. Cell Death Dis..

[23] Gerami P (2017). Development and validation of a noninvasive 2-gene molecular assay for cutaneous melanoma. J. Am. Acad. Dermatol..

[24] Tay Y, Rinn J, Pandolfi PP (2014). The multilayered complexity of ceRNA crosstalk and competition. Nature.

[25] Gao W (2017). MicroRNA-204-5p inhibits invasion and metastasis of laryngeal squamous cell carcinoma by suppressing forkhead box C1. J. Cancer.

[26] Luan W (2017). miR-204-5p acts as a tumor suppressor by targeting matrix metalloproteinases-9 and B-cell lymphoma-2 in malignant melanoma. OncoTargets. Ther..

[27] Wang X, Li F, Zhou X (2016). miR-204-5p regulates cell proliferation and metastasis through inhibiting CXCR4 expression in OSCC. Biomed. Pharmacother. = Biomedecine pharmacotherapie.

[28] Yin Y (2014). miR-204-5p inhibits proliferation and invasion and enhances chemotherapeutic sensitivity of colorectal cancer cells by downregulating RAB22A. Clin. Cancer Res..

[29] Luan W (2018). miR-137 inhibits glutamine catabolism and growth of malignant melanoma by targeting glutaminase. Biochem. Biophys. Res. Commun..

[30] Liu H, Dai C, Wu Q, Liu H, Li F (2017). Expression profiling of long noncoding RNA identifies lnc-MMP3-1 as a prognostic biomarker in external auditory canal squamous cell carcinoma. Cancer Med..

[31] Subramanian M, Li XL, Hara T, Lal A (2015). A biochemical approach to identify direct microRNA targets. Methods Mol. Biol..

[32] Luan W (2015). PKM2 promotes glucose metabolism and cell growth in gliomas through a mechanism involving a let-7a/c-Myc/hnRNPA1 feedback loop. Oncotarget.

