# Supplementary material for: Long noncoding RNA LINC00518 acts as a competing endogenous RNA to promote the metastasis of malignant melanoma via miR-204-5p/AP1S2 axis
Source: Cell Death Dis. 2019 Nov 11;10(11):855. doi: 10.1038/s41419-019-2090-3 (PMC6848151; doi:10.1038/s41419-019-2090-3)
Supplement: Supplementary file 1 — Experimental Method of Supplementary Materials [file 41419_2019_2090_MOESM1_ESM.doc]

**Methods**

Cell proliferation assay and flow cytometry assay

For the CCK-8 (Beyotime, China) assay, transfected melanoma cells were added to 96-well plates, and the medium of each well was replaced with 100 μl culture media containing 10% CCK-8 at different times (12, 24, 36 and 48 h). The absorbance was measured using a microplate reader (Multiscan FC, Thermo Scientific) at an optical density of 450 nm. The melanoma cell apoptosis ratio were measured using flow cytometry. Annexin V-FITC apoptosis detection kit (BD Biosciences, USA) was used to analyze the apoptosis ratio, and the percentages of apoptosis was evaluated using Annexin V-FITC and propidium iodide (PI) double staining.
