# Supplementary figures and images for: Long noncoding RNA LINC00518 acts as a competing endogenous RNA to promote the metastasis of malignant melanoma via miR-204-5p/AP1S2 axis
Source: Cell Death Dis. 2019 Nov 11;10(11):855. doi: 10.1038/s41419-019-2090-3 (PMC6848151; doi:10.1038/s41419-019-2090-3)

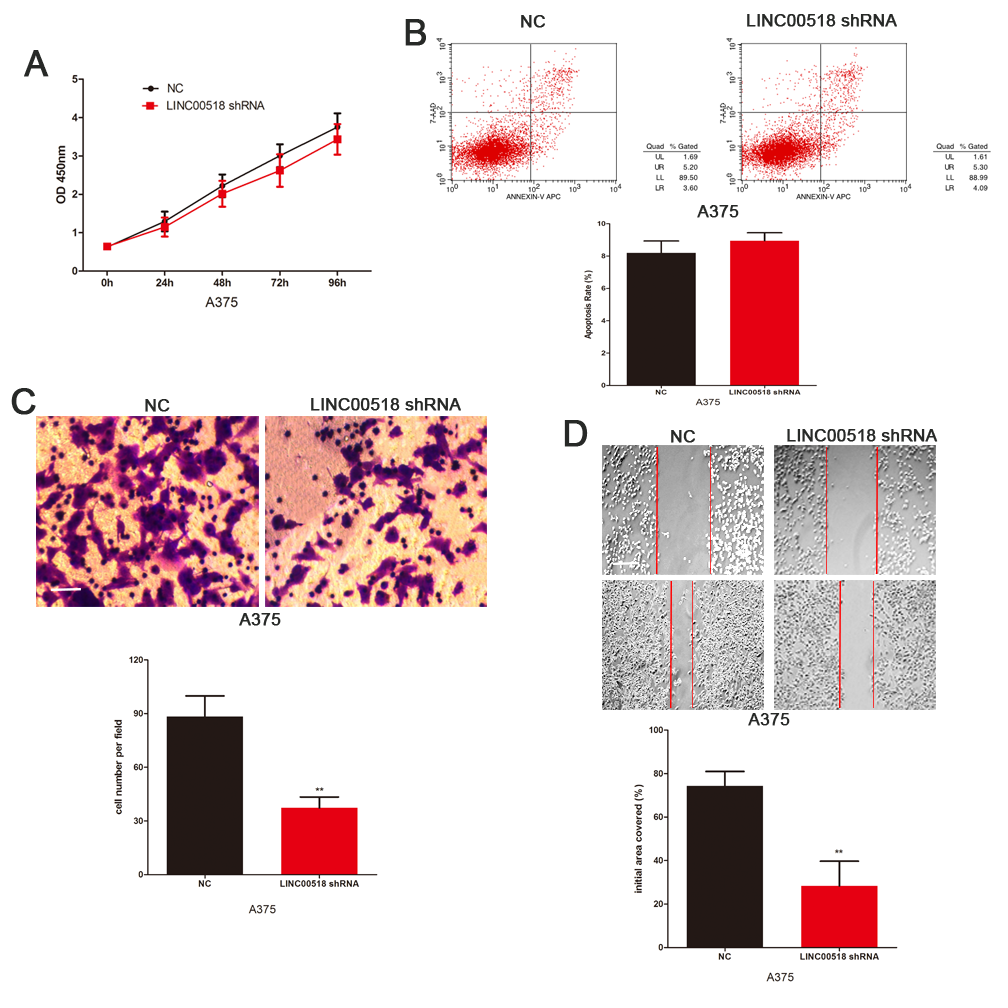

Supplement: Supplementary file 2 — Supplementary Figure 1 [file 41419_2019_2090_MOESM2_ESM.tif]

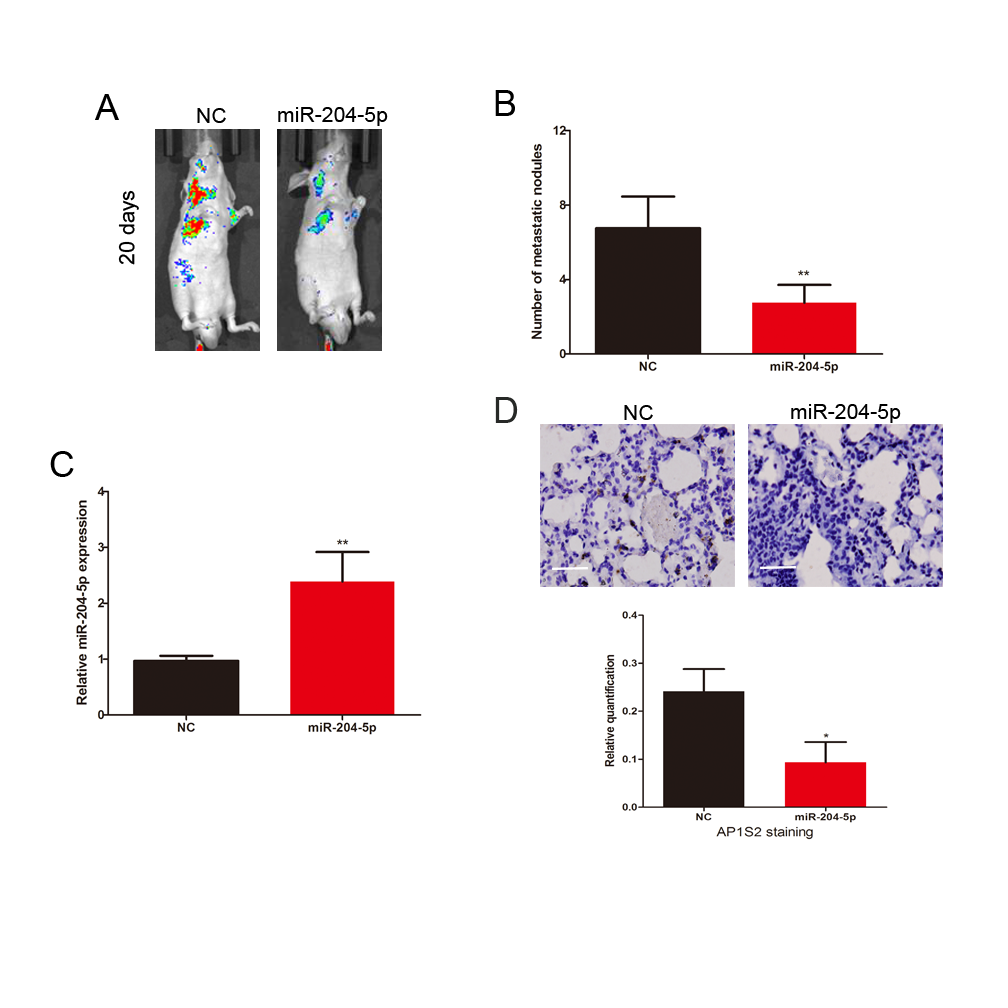

Supplement: Supplementary file 3 — Supplementary Figure 2 [file 41419_2019_2090_MOESM3_ESM.tif]
